# How to See a Post-Nasal Space

**Published:** 1908-06-13

**Authors:** 


### THE GENERAL PRACTITIONER'S COLUMN.

[Contributions to this Column are invited, and if accepted will be paid for.]

### HOW TO SEE A POST-NASAL SPACE.

In all work with reflected light the first essential is to acquire the habit of automatically keeping the area under examination flooded with the greatest possible intensity of illumination. In no case is this more important than when one is dealing with the recesses of the naso-pharynx. The lamp (at the patient's left hand) is at the same level as one's head-mirror, and so directed that one takes the light from its centre, not from its edge. Looking with the right eye through the hole in the mirror, one directs light on to the patient's forehead, and approaches, or recedes from, him till a focus is obtained. He is then directed to open his mouth, and at the distance thus found the posterior pharyngeal wall is illuminated. The patient's head should be slightly higher than one's own: small children may sit on a box placed on the chair. The next thing is to control the tongue, and to do this properly is half the battle. The best instrument is a simple half-inch strip of metal bent at slightly less than a right angle. The vertical limb is held in the palm, the thumb on top of the bend of the instrument, and the knuckles and backs of the fingers resting against the patient's chin, with the little finger, placed under it, steadying the whole head. The end of the horizontal portion is placed rather further back than half way along the tongue, which, with a light, rather quick movement, effected by lateral flexion of the wrist and supination of the forearm, is depressed, and at the same time drawn forwards. A tongue should never be depressed but with the left hand, which will then soon get the necessary practice. Forcible pressure is likely to produce "gagging," but, on the other hand, at least an inch of the posterior pharyngeal wall must be exposed, or else it is probably useless to insert the mirror.

This latter feat needs some finesse. One directs the patient to go on breathing—through the nose if possible—and inserts a warmed No. 2 mirror, held pen-fashion, into the widely opened mouth. Before it has got far the shaft should be steadied against the left corner of the mouth or a neighbouring tooth. This helps one to manœuvre the mirror into the mid-line, as low and as far back as possible, without touching anything. Spasm may often be checked by telling the patient he is "doing capitally," or by directing him to sniff sharply through the nose or to look at one's face. With the mirror in position and the parts before mentioned or the base of the tongue as a fulcrum, the handle is twisted between the fingers, inclining the mirror so as to reflect the different structures: those below the levels of the middle turbinates may be seen by cautiously raising the mirror a little. It is then withdrawn quickly, and before the patient shows any signs of discomfort. After a rest a second inspection completes the examination.

